# Impact of an Age-Stratified Augmented Take-Home Ration on Nutritional Recovery in Under-Five Children With Uncomplicated SAM: A Community-Based Prospective Study in Southern Jharkhand

**DOI:** 10.7759/cureus.116521

**Published:** 2026-09-19

**Authors:** Shalini Sundram, Anit Kujur, Manisha Kujur, Kumari Asha Kiran, Vidya Sagar, Hirendra Birua

**Affiliations:** 1 Community Medicine, Rajendra Institute of Medical Sciences, Ranchi, IND; 2 Preventive Medicine, Rajendra Institute of Medical Sciences, Ranchi, IND; 3 Pediatric Surgery, Rajendra Institute of Medical Sciences, Ranchi, IND

**Keywords:** community-based management of acute malnutrition, nutritional recovery, severe acute malnutrition, shishu shakti, take-home ration

## Abstract

Background: Community-based management of acute malnutrition (CMAM) often lacks context-specific evidence regarding the scaling and age-stratification of Take-Home Rations (THR). This study evaluates the association of nutritional recovery at four-month follow-up with this age-appropriate augmented THR ("Shishu Shakti"), delivering 490 kcal and 17 g of protein per 100 g versus standard uniform THR, which yielded 432.27 kcal and 13.95 g of protein per 100 g among children with uncomplicated severe acute malnutrition (SAM) in Southern Jharkhand.

Methods: A community-based prospective cohort study enrolled 205 children aged 6-59 months with uncomplicated SAM. Group A (n= 139) received the age-stratified augmented Shishu Shakti THR (90-160 g/day), while Group B (n = 66) received the uniform standard THR (180 g/day). Anthropometric parameters and nutritional classifications were tracked over a four-month longitudinal follow-up.

Results: Significant baseline disparities existed between cohorts: Group A children were younger (26.85 ± 14.41 months vs. 35.48 ± 15.33 months; t = -3.924, p < 0.001) and presented with lower baseline weight (7.71 ± 2.03 kg vs. 9.26 ± 2.29 kg; t = -4.899, p < 0.001). At the four-month follow-up, Group A achieved a significantly higher nutritional recovery rate (75.5%, n = 105) compared to Group B (60.6%, n = 40) (χ2 = 11.15, p = 0.011). Persistent Moderate Acute Malnutrition (MAM) at discharge was lower in Group A (13.7%, n = 19) than in Group B (33.3%, n = 22). Age-stratified tracking confirmed superior normalization across all younger age tiers in the intervention cohort.

Conclusion: Children receiving the age-stratified augmented THR had a higher crude proportion of nutritional recovery at four months than children receiving the standard uniform THR.

## Introduction

Jharkhand continues to report high childhood malnutrition rates according to NFHS-5, with elevated levels of stunting, wasting, and underweight among children under five [1]. Community-based management of severe acute malnutrition (SAM) is essential to reach vulnerable and hard-to-reach populations, reduce child mortality, and improve development outcomes [2]. Alongside active screening, medical care, and counseling, access to energy-dense foods is a critical component of community-based treatment. The World Health Organization recommends ready-to-use therapeutic food (RUTF) for the management of uncomplicated SAM to support anthropometric recovery, but the applicability of WHO food standards to the Indian context is debated [3]. The National Community-based Management of Acute Malnutrition (CMAM) Guideline (October 2023) specifies the macro- and micronutrient contributions expected from supplementary nutrition (take-home ration (THR) or hot-cooked meals) combined with home food [4]. Recommended energy and protein targets for undernourished children are 400 kcal and 15-20 g of protein for children aged 6-12 months, 700 kcal and 25-30 g of protein for those aged 1-3 years, and 800 kcal and 25-30 g of protein for those aged 3-6 years [5].

In Jharkhand’s current community SAM program for children aged 6-59 months, all children receive six packets of THR (Shishu Ahaar or Poshtik Meetha Daliya; 180 g) intended to last 25 days, with a uniform daily serving of 180 g irrespective of age or weight [6]. This standard THR does not meet the higher nutrient density required for SAM children, particularly in younger age groups, and necessitates large quantities that may be impractical for infants. In response, a State THR Committee, including experts from the National Institute of Nutrition (Hyderabad), Birsa Agricultural University (Ranchi), the Centre of Excellence for SAM Management (RIMS), Ranchi, and UNICEF (Jharkhand), revised the THR formulation.

The revamped Shishu Shakti Take Home Ration (augmented take-home ration (ATHR)) initiative was launched as a pilot project in West Singhbhum district by the Women, Child Development, and Social Security Department. The formulation per serving includes whole wheat flour (10 g), roasted ragi flour (5 g), roasted Bengal gram flour (15 g), roasted groundnut (20 g), powdered sugar (10 g), soybean oil (15 g), milk powder (15 g), processed soya flour (9 g), and a vitamin-mineral mix [7]. The present study aims to evaluate the association between nutritional recovery after four months of follow-up and consumption of this age-appropriate augmented THR (Shishu Shakti) compared with standard uniform THR among uncomplicated SAM children under five years of age in West Singhbhum district, Jharkhand.

## Materials and methods

Study design

This was a community-based prospective cohort pilot study.

Study duration

The study was carried out over a six-month period from January to June 2025.

Study site

The ATHR group was located within the Chakradharpur Block Integrated Child Development Services (ICDS) project area in West Singhbhum district, where eligible children received the augmented THR under the Community-Based Management of Acute Malnutrition (CMAM) program. The Bandgaon block of West Singhbhum district served as the control site, where children received the standard uniform THR under the same CMAM framework.

Ethical consideration

The study was initiated following formal approval from the Institutional Ethics Committee of the Rajendra Institute of Medical Sciences (RIMS) (Memo No. 268).

Study participants

The study population consisted of children aged 6-59 months diagnosed with uncomplicated SAM and enrolled in the local CMAM program. Written informed consent was obtained from the parents or primary caregivers prior to enrollment. Exclusion criteria applied to infants under six months of age, children continuing CMAM care after being discharged from a Malnutrition Treatment Centre (MTC) at the time of study initiation, and children presenting with complicated SAM (defined by the presence of bilateral pitting edema or a failed appetite test).

Sample size

Sample size was estimated using preliminary screening and growth monitoring drive across all 10 sectors of Chakradharpur block, identifying 385 children with SAM, representing a localized prevalence of 2.7% among children aged 6-59 months. As this was a pilot study, the sample size was planned pragmatically rather than through a formal power calculation. Convenience sampling was done, and we approached all 385 eligible children, but only 139 children whose parents were willing to participate were successfully enrolled in the ATHR group (Group A) after obtaining parental consent. Similarly, in the control block of Bandgaon, 66 children whose parents were willing to participate were enrolled in the standard uniform THR cohort (Group B) based on parental consent.

To ensure age-wise representation across both cohorts, the sample was stratified into three distinct age intervals: 6-12 months, 13-36 months, and 37-59 months. The ATHR (Group A) doses are 90, 150, and 160 g/day, while the standard uniform THR (Group B) receives 180 g/day.

The augmented Shishu Shakti (ATHR) formulation provided 490 kcal and 17 g protein per 100 g. Based on the prescribed age-specific quantities, children aged 6-12 months received approximately 441 kcal and 15.3 g protein/day from 90 g of THR; those aged 13-36 months received approximately 735 kcal and 25.5 g protein/day from 150 g; and those aged 37-59 months received approximately 784 kcal and 27.2 g protein/day from 160 g. In comparison, children receiving the standard uniform THR were prescribed 180 g/day, corresponding to approximately 778 kcal and 25.1 g protein/day based on the reported nutrient composition of 432.27 kcal and 13.95 g protein per 100 g.

Thus, although the augmented formulation had greater nutrient density per 100 g, the total daily energy and protein supplied were not uniformly higher than the standard ration across all age groups. Ration adherence and actual consumption were assessed using the Suposhan card provided to mothers, who recorded consumption daily after each feed. Ration adherence and actual consumption were assessed using the Suposhan card provided to mothers, who recorded consumption daily after each feed. Mothers were trained and were demonstrated how to correctly mark the card to ensure accurate recording at the time of THR distribution.

Data collection tools

Data were captured using semi-structured questionnaires developed and deployed via KoboCollect (Kobo, Cambridge, Massachusetts).

Data Collection Technique

Field operations were executed by a dedicated research team consisting of four block coordinators and one district coordinator. Each child was tracked fortnightly over a longitudinal four-month observation window, totalling a maximum of eight follow-up assessments. These follow-ups were carried out through alternating Anganwadi Center evaluations and structured home visits. Anthropometric metrics and nutritional classifications (SAM, moderate acute malnutrition (MAM), Normal, and Defaulter) were tracked over a four-month longitudinal follow-up. As per the National Health Systems Resource Centre (NHSRC), which provides technical support to the National Health Mission, a child is classified as having SAM if the weight-for-height/length is below -3 SD, or there is bilateral pitting edema. MAM is diagnosed when the weight-for-height/length falls between -2 SD and -3 SD, without edema. Nutritional recovery was considered when the child achieves weight-for-height/length to -2 SD or above in the absence of edema [8]. Field quality control, procedural adherence, and anthropometric verification were supervised via routine monitoring visits conducted by State SCoE (State Centre of Excellence) coordinators, senior faculty members, and postgraduate residents from the Department of Community Medicine, Rajendra Institute of Medical Sciences, Ranchi.

The primary outcome was the nutritional recovery among children with uncomplicated SAM under the ATHR regimen compared to those on the standard uniform protocol. Secondary variables included sociodemographic characteristics, dietary and infant feeding practices, health service utilization, and programmatic implementation barriers. For the present analysis, nutritional recovery was defined as the transition from SAM at enrollment to normal nutritional status at the final four-month assessment, based on the study's anthropometric classification system. Normal nutritional status was defined as weight-for-height/length z-score (WHZ/WLZ) ≥ -2 SD in the absence of bilateral pitting oedema. MAM was defined as WHZ/WLZ between -3 SD and -2 SD without edema, while SAM was defined as WHZ/WLZ < −3 SD and/or bilateral pitting edema. Admission classification was based on the anthropometric, appetite test, and clinical assessment performed at enrollment into the community-based management program. Children with bilateral pitting edema or failed appetite testing were excluded and referred to the malnutrition treatment center, as having complicated SAM. At the final assessment, nutritional status was classified using weight-for-height/length z-score.

WHO growth standards were used for calculation of weight-for-height/length z-scores (WHO Anthro Plus software version 1.0.4). Children who migrated before the final assessment were classified as migrated/withdrawn and were not considered nutritionally recovered.

Statistical analysis

All data were cleaned, entered into MS Excel (Microsoft Corporation, Redmond, Washington), and analyzed using IBM SPSS Statistics for Windows, Version 20 (Released 2011; IBM Corp., Armonk, New York). Categorical variables were expressed as frequencies and percentages, while continuous anthropometric metrics were summarized using means and standard deviations (± SD) for the categorical variables. Pearson's chi-square test was used for categorical values, and independent t-tests were used to evaluate significant mean differences between the two independent study groups. Multivariable logistic regression was applied to generate the adjusted odds ratios (AOR), 95% confidence intervals, and adjusted p-values. Statistical significance was defined a priori at a p-value of less than 0.05.

## Results

The study evaluated a total cohort of 205 under-five children diagnosed with uncomplicated SAM, comprising 139 children in the ATHR (Group A) and 66 children in the standard uniform THR (Group B). Gender distribution was statistically comparable between the two arms (X^2^ = 0.982, p = 0.322), with males constituting 64.7% (n = 90) of Group A and 57.6% (n = 38) of Group B (Table 1).

**Table 1:** Sociodemographic profile and baseline homogeneity of the enrolled severe acute malnutrition (SAM) cohort. Group A = children enrolled in the ATHR group (n = 139); Group B = children enrolled in the standard uniform THR group (n = 66). * indicates statistical significance at p < 0.05. The chi-square test was used for univariate analysis, and for adjusted analysis accounting for age, sex, and ethnicity, multivariate logistic regression was used. Age was entered as a continuous variable in the multivariable logistic regression; therefore, the AOR 0.744 represents the change in odds of recovery for each additional year of age. ATHR: augmented take-home ration; THR: take-home ration; AOR: adjusted odds ratio.

Variable		Group A (n = 139)	Group B (n = 66)	Chi-square value (χ2)	p-value	Adjusted odds ratio	95% CI	p-value
Gender, n (%)	Male	90 (64.7%)	38 (57.6%)	0.982	0.322	Reference		
	Female	49 (35.3%)	28 (42.4%)			0.940	0.581-0.954	0.020*
Ethnicity, n (%)	Tribal	119 (85.6%)	2 (3.0%)	126.186	<0.001*	Reference		
	Non-tribal	20 (14.4%)	64 (97.0%)			190.40	43.13-840.60	<0.001*
Age distribution, n (%)	6-11 months	16 (11.5%)	5 (7.6%)	15.369	0.004*	Reference		
	12-23 months	54 (38.9%)	11 (16.7%)			1.520	0.485-4.764	0.478
	24-35 months	28 (20.1%)	19 (28.8%)			2.150	0.682-6.782	0.189
	36-47 months	23 (16.5%)	12 (18.2%)			1.840	0.559-6.058	0.315
	48-59 months	18 (13%)	19 (28.8%)			2.850	0.884-9.186	0.080
Age (per one-year increase)						0.744	0.581-0.954	0.020*

Statistically significant baseline disparities were observed between the study cohorts in ethnic background and age structure (Table 1). A marked ethnic divergence was evident (X^2^ = 126.186, p < 0.001), with the vast majority of children in Group A belonging to tribal communities (85.6%, n = 119), whereas Group B was predominantly non-tribal (97.0%, n = 64), yielding an AOR of 190.40 with a wide confidence interval (95% CI: 43.13-840.60, p = 0.001), which primarily indicates high statistical uncertainty and a lack of precision regarding the true population parameter, meaning the actual effect could range from negligible to substantial. This imprecision prevents researchers from drawing definitive conclusions about the ethnic background. Furthermore, the age category distribution differed significantly across the groups (X^2^ = 15.369, p = 0.004), with Group A exhibiting a higher concentration of infants and young toddlers aged 12-23 months (38.9% vs. 16.7%). Multivariable regression analysis further demonstrated that age (per one-year increase) was an independent predictor associated with the outcome (AOR = 0.744, 95% CI: 0.581-0.954, p = 0.020) (Table 1). Consequently, the mean age at admission was significantly lower in Group A (26.85 ± 14.41) than in Group B (35.48 ± 15.33 months, t = -3.924, p < 0.001; 95% CI: -12.97 to -4.29) (Table 2). Reflecting this younger baseline profile, children in Group A presented with significantly lower admission body weight (7.71 ± 2.03 kg vs. 9.26 ± 2.29 kg, t = -4.899, p < 0.001; 95% CI: -2.20 to -0.90) and shorter recumbent length/height (78.20 ± 10.18 vs. 90.95 ± 13.83, t = -7.432, p < 0.001; 95% CI: -16.13 to -9.37) (Table 2).

**Table 2:** Baseline demographics and clinical characteristics at admission. The ATHR group (Group A) comprised 139 children, while the standard uniform THR group (Group B) comprised 66 children. Comparisons between groups were made using the independent samples t-test. * indicates statistical significance at p < 0.05. ATHR: augmented take-home ration; THR: take-home ration.

Parameter	Group A (n = 139)	Group B (n = 66)	t-value	p-value	95% confidence interval of the difference
Age (months), mean ± SD	26.85 ± 14.41	35.48 ± 15.33	-3.924	<0.001*	-12.97 to -4.29
Weight (kg), mean ± SD	7.71 ± 2.03	9.26 ± 2.29	-4.899	<0.001*	-2.20 to -0.90
Height (cm), mean ± SD	78.20 ± 10.18	90.95 ± 13.83	-6.892	<0.001*	-16.13 to -9.37

At final exit assessment, mean anthropometric parameters were 9.46 ± 2.04 in weight and 78.95 ± 9.92 in height for Group A, compared to 11.26 ± 2.28 and 92.62 ± 13.81 for Group B (p < 0.001 for both comparisons) (Table 3).

**Table 3:** Anthropometric outcomes at discharge. Group A = children enrolled in the ATHR Group (n = 139); Group B = children enrolled in the standard uniform THR Group (n = 66). “At discharge” refers to the anthropometric measurements recorded at the time the child met the study’s discharge/recovery criteria. Comparisons between groups were made using the independent samples t-test. * indicates statistical significance at p < 0.05. ATHR: augmented take-home ration; THR: take-home ration.

Parameter	Group A (n = 139)	Group B (n = 66)	t-value	p-value	95% confidence interval of the difference
Discharge weight (kg), mean ± SD	9.46 ± 2.04	11.26 ± 2.28	-7.312	<0.001*	-3.126 to -1.797
Discharge height (cm), mean ± SD	78.95 ± 9.92	92.62 ± 13.81	-7.606	<0.001*	-17.720 to -10.416

At the conclusion of the four-month longitudinal follow-up, the primary clinical outcome differed significantly between the two therapeutic regimens (X^2^ = 11.15, p = 0.011) (Table 4). In Group A, 75.5% (n = 105) achieved complete nutritional recovery (transition to normal nutritional status), compared to 60.6% (n = 40) in Group B (Table 4). Only 13.7% (n = 19) of children in Group A remained in the MAM stage at discharge, compared to 33.3% (n = 22) in Group B (Table 4). Persistent SAM at exit was minimal across both cohorts (2.9%, n = 4 in Group A vs. 1.5%, n = 1 in Group B), and program attrition due to migration was recorded at 7.9% (n = 11) and 4.5% (n = 3), respectively (Table 4).

**Table 4:** Nutritional outcomes of the enrolled SAM cohort at a four-month follow-up. Group A = children enrolled in the ATHR Group (n = 139); Group B = children enrolled in the standard uniform THR Group (n = 66). * indicates statistical significance at p < 0.05. MAM: moderate acute malnutrition; SAM: severe acute malnutrition; ATHR: augmented take-home ration; THR: take-home ration.

Nutritional status at discharge	Group A (n = 139)	Group B (n = 66)	Chi-square value (X^2^)	p-value
Normal, n (%)	105 (75.5%)	40 (60.6%)	11.15	0.011*
MAM, n (%)	19 (13.7%)	22 (33.3%)		
SAM, n (%)	4 (2.9%)	1 (1.5%)		
Migrated, n (%)	11 (7.9%)	3 (4.5%)		
Total, n (%)	139 (100.0%)	66 (100.0%)		

Age-stratified longitudinal tracking demonstrated that recovery proportions varied across age categories. Group A had higher recovery proportions in the 6-11, 12-23, 24-35, and 48-59 months categories, whereas Group B showed a higher crude recovery proportion in the 36-47 months category. The four-month follow-up demonstrates that Group A achieved a higher overall recovery proportion (75.50%, 105/139) compared to Group B (60.60%, 40/66), yielding a statistically significant risk difference of +14.93% (95% CI: 1.1% to 28.7%) and an odds ratio of 2.00 (95% CI: 1.02-3.93). While recovery rates were comparable between the cohorts in the youngest age bracket (6-11 months), Group A consistently outperformed Group B across most subsequent age categories, with the most pronounced difference observed in the 24-35 months group (75.00% vs. 47.37%) (Figure 1).

**Figure 1:**
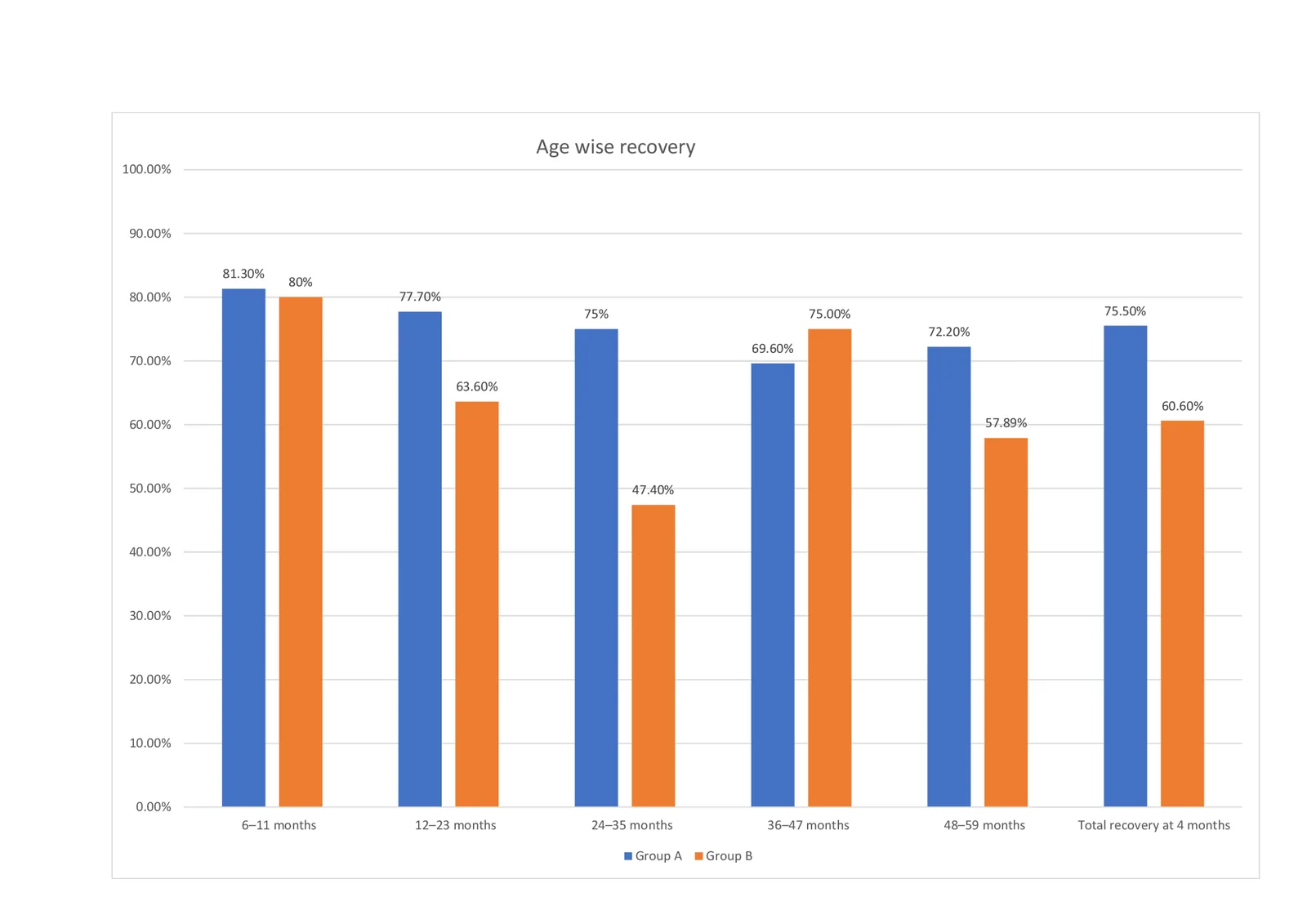
Distribution of recovery age wise among both the groups. Group A = children enrolled in the ATHR Group (n = 139); Group B = children enrolled in the standard uniform THR Group (n = 66). 6-11 months: Both cohorts exhibit high and comparable recovery proportions, with Group A at 81.30% (13/16) and Group B at 80.00% (4/5). 12-23 months: Group A maintains a strong recovery rate of 77.70% (42/54), whereas Group B drops to 63.60% (7/11). 24-35 months: Recovery in Group A stands at 75.00% (21/28), while Group B experiences a noticeable decline to 47.40% (9/19). 36-47 months: Group B shows a temporary peak at 75.00% (9/12), slightly surpassing Group A's rate of 69.60% (16/23). 48-59 months: Group A recovers to 72.20% (13/18), outperforming Group B's 57.89% (11/19). Total recovery at four months: Across all age groups combined, Group A achieves an overall recovery proportion of 75.50% (105/139), which is markedly higher than Group B's overall recovery proportion of 60.60% (40/66). ATHR: augmented take-home ration; THR: take-home ration.

## Discussion

This community-based prospective cohort study observed a higher crude proportion of nutritional recovery at four months among children receiving the age-stratified augmented Shishu Shakti THR compared with children receiving the standard uniform THR. Recovery to normal nutritional status was observed in 75.5% of children in Group A and 60.6% in Group B, while persistent MAM was less frequent in Group A.

The 75.5% recovery rate achieved by the ATHR arm meets the international SPHERE benchmark (>75%) for therapeutic feeding programs managing SAM [9]. These findings align closely with CMAM initiatives across India. Burza et al. [10] documented high cure rates and minimal mortality using structured community protocols in Bihar. Similarly, Satav et al. [11] reported strong catch-up growth and nutritional normalization among severely undernourished tribal children in rural Maharashtra using locally prepared, energy-dense therapeutic foods combined with close community follow-up.

In contrast, the lower recovery rate (60.6%) and high proportion of persistent MAM in Group B mirror the challenges identified by Patel [12], who reported a cure rate of only 44.8% after 12 weeks of community management, with nearly half of the children failing to attain full recovery. Patel observed that older baseline age, larger gastric volume requirements, and intra-household sharing significantly impair recovery under flat-rate rations [13]. This explains the slower response in Group B, whose mean age was significantly higher (35.48 ± 15.33 months). Flat-rate rations of 180 g/day impose substantial bulk restrictions on younger infants and fail to deliver adequate age-matched micronutrient density to toddlers, whereas the scoop-calibrated Shishu Shakti formulation delivers 490 kcal and 17 g protein per 100 g in manageable feeding volumes.

Younger children present heightened vulnerability to wasting due to rapid metabolic turnover and infectious episodes, yet retain greater plasticity for catch-up growth when provided nutrient-dense therapeutic nutrition [14,15]. The success of Shishu Shakti within the operational framework of Anganwadi Centers confirms the feasibility and cost-effectiveness of local, decentralized production models over imported commercial formulations. Moreover, the low overall attrition rate (6.8% combined default/migration) confirms strong community acceptability and adherence. Because baseline age and anthropometry differed markedly between cohorts, future multi-center evaluations should incorporate weight velocity metrics (g/kg/day) and longitudinal WHO Z-score trajectories to isolate intervention effects from physiological maturation [15].

This study has a few limitations. First, its non-randomized controlled design resulted in baseline age and ethnic disparities between cohorts, which may introduce confounding. Second, the four-month follow-up period restricts the assessment of long-term nutritional sustainability and post-intervention relapse rates. Third, potential intra-household sharing of rations and reliance on self-reported compliance could affect true intake accuracy. Finally, the interpretation of these study outcomes is limited by several significant methodological constraints, including unequal group sample sizes, geographic confounding from distinct study sites, and pronounced baseline differences in age and ethnicity. Furthermore, extreme data sparsity resulted in quasi-complete separation, restricting the reliability of multivariable adjustments and indicating that residual confounding likely influences the observed differences.

## Conclusions

Children receiving the age-stratified augmented “Shishu Shakti” (ATHR) had a higher crude proportion of nutritional recovery at four months than children receiving the standard uniform THR. However, substantial baseline and site-level differences between cohorts limit causal interpretation. Controlled studies with appropriate adjustment for baseline nutritional status and other potential confounders are warranted.
